# Role of connexin 32 in the directional differentiation of induced pluripotent stem cells into hepatocytes

**DOI:** 10.7150/ijms.83973

**Published:** 2024-01-12

**Authors:** Yan Xu, Yufeng Wang, Ran Qi, Kun Li, Xiuyan Wang, Xinbo Li, Baomin Shi

**Affiliations:** 1 Department of General Surgery, Tongji Hospital, School of Medicine, Tongji University, Shanghai, 200065, China.; 2 Department of Ultrasonography, Tongji Hospital, School of Medicine, Tongji University, Shanghai, 200065, China.; 3 Ophthalmology, Casey Eye Institute, Oregon Health & Science University, Portland, Oregon, 97239, USA.; 4 Department of General Surgery, Xinhua Hospital, School of medicine, Shanghai Jiaotong University, 200025, China.

**Keywords:** Connexin32, induced pluripotent stem cells, hepatocyte differentiation, urine-derived epithelial cell, regulatory factor

## Abstract

This study aimed to explore the role of connexin 32 (Cx32) in the directional differentiation of induced pluripotent stem cells (iPSCs) into hepatocytes. Urine-derived epithelial cells were collected from the fresh urine of a healthy donor and transducted with reprogramming plasmid mixture to generate iPSCs. The iPSCs were then directionally differentiated into hepatocytes. During the differentiation, the upregulated and downregulated groups were treated with vitamin K2 (VK2) and 2-aminoethoxyboronate diphenylester (2-APB) to increase and inhibit Cx32 expression, respectively. The control group was not treated with the regulatory factor. Expression of Cx32 and hepatocyte-specific markers, including AFP, hepatocyte nuclear factor 4α (HNF-4α), albumin (ALB) and cytokeratin 18 (CK18) were detected. It indicated that Cx32 expression was not observed in iPSCs, but gradually increased during the process of hepatic differentiation from iPSCs. Upregulation of Cx32 expression by VK2 treatment promoted hepatocyte maturation and enhanced the expression of the aforementioned hepatic specific markers, whereas downregulation of Cx32 expression by 2-APB treatment had the opposite effects. In conclusion, urine-derived iPSCs could be directionally differentiated into hepatocytes. Up-regulation of Cx32 improves the efficiency and maturity of differentiation of iPSCs into hepatocytes, and Cx32 may be a promoting factor during the process of hepatic differentiation from iPSCs.

## Introduction

Acute liver failure (ALF) is a severe consequence of abrupt hepatocyte injury and has a high mortality rate 1. Chronic liver failure is also a life-threatening condition and may evolve as end-stage liver disease (ESLD) and/or cancer, resulting in high rates of mortality 2. The only curative treatment for both ALF and ESLD is liver transplantation, the application of which has been impeded by the shortage of donors 3. Since hepatocytes can repair and replace the host liver, transplantation of hepatocytes has been revealed as an alternative treatment strategy to liver transplantation. However, potential issues include the donor shortage, and that hepatocytes are difficult to preserve and exhibit low proliferation in cultures 4. Generation of fully functional hepatocytes from stem cells has become a promising available approach for hepatocyte transplantation.

Pluripotent stem cells have been widely investigated for their promising medical use in regenerative medicine 5. Induced pluripotent stem cells (iPSCs) are a type of pluripotent stem cells be generated from somatic cells by reprogramming them to a pluripotent state, which is capable of differentiating into various cell types, including hepatocytes. Several studies have recovered the procedure of differentiating iPSC into hepatocytes 6,7. Four transcription factors, including forkhead box A1, forkhead box A3, CCAAT enhancer binding protein α and CCAAT enhancer binding protein β, have been reported to serve a key role in cell fate reprogramming, then contributing to direct differentiation of human iPSCs into hepatocytes 8. However, the mechanisms underlying the differentiation of iPSCs into hepatocytes have not been fully elucidated.

Connexin is a component of gap junctions, which has been demonstrated to mediate intercellular activities of gap junctional intercellular communication (GJIC) 9. Furthermore, more than 20 kinds of connexins have been identified in humans and most cell types express more than one type of connexin 10. Connexins and GJIC are involved in stem cell proliferation and differentiation 11-13. Diverse types of connexins, including connexin 32 (Cx32) and connexin 26 (Cx26), are found in well-organized tissue of the adult liver, and these construct a GJIC network between hepatocytes and are essential for functional differentiation 14. Notably, it has been reported that overexpression of Cx32 promotes the differentiation of hepatic progenitor cells into hepatocytes 15. Cx32-mediated cell-cell communication serves a crucial role in hepatic differentiation from human embryonic stem cells (hESCs) 16. However, to the best of our knowledge, the role of Cx32 in the directional differentiation of iPSCs into hepatocytes has not been reported.

In the present study, iPSCs were generated from urine-derived renal tubular epithelial cells and then directionally differentiated into hepatocytes. The objective of the present study was to explore the role of Cx32 in the directional differentiation of iPSCs into hepatocytes. It was revealed that upregulation of Cx32 expression by vitamin K2 (VK2) treatment promoted hepatocyte maturation and enhanced the expression of hepatic specific markers, whereas downregulation of Cx32 expression by 2-aminoethoxyboronate diphenylester (2-APB) treatment had the opposite effects. The findings will provide a preliminary research basis for optimizing and improving the differentiation efficiency of iPSCs into hepatocytes.

## Materials and methods

*Urinary cell isolation.* The study was approved by the Ethics Committee of Tongji Hospital Affiliated to Tongji University, Shanghai, China. Urine samples were collected from a 26-year-old healthy adult woman without urinary and renal disease. And the donor's written consent was obtained after understanding the purpose of all the procedures. Fresh middle urine (200 ml) was collected, and centrifuged at 800 x g for 5 min in room temperature. Subsequently, the cell pellets were resuspended and washed with 10 ml cold PBS. Centrifuged again, the supernatant was discarded, and the pellets were resuspended with 1 ml urine cell medium [UCM; DMEM/F12 (STEMCELL Technologies, catalogue number#36254) containing 10% FBS (MilliporeSigma), 0.1 mM non-essential amino acid, 0.1 mM β-mercaptoethanol and 1 mM Glutamax + SingleQuot (Lonza Group, Ltd.)]. Cell suspension was transferred to a six-well plate precoated with 0.1% gelatin in ultra-pure water for 30 min, and then incubated with 4% CO_2_ for 24 h at 37˚C. For the first 3 days, 1 ml fresh UCM was added every day. On day 4, 3 ml medium was sucked out and 1 ml fresh UCM was added. Subsequently, half of the medium was replaced with fresh medium each day until the first colony appeared. When the cell fusion rate reached 80-90%, the cells were released by incubation with trypsin/EDTA (Stemcell Technologies, Inc.) for 2 min. The defined trypsin inhibitor (STEMCELL Technologies, Inc.) was then added to terminate the trypsin reaction. urinary derived epithelial cells, which mainly comprised renal tubular epithelial cells were then harvested by centrifugation at 800 x g for 5 min in room temperature.

*Generation of iPSCs from urinary derived epithelial cells.* urinary derived epithelial cells (1x10^6^ cells/0.1 ml) were collected and then transduced with the reprogramming plasmid mixture encoding OCT4, SOX2, LIN28(a kind of RNA-binding protein), Kruppel like factor 4, L-MYC, p53short hairpin RNA and microRNA-302/367 clusters using a Episomal iPSC Reprogramming Plasmid kit according to manipulation (System Biosciences, LLC) and a Lonza 4D electrotransfection apparatus. The aforementioned cells were then plated into each well of Matrigel-coated six-well culture dishes with UCM. After 24 h of incubation, the UCM was replaced with the mTeSR1 medium (STEMCELL Technologies, Inc. catalogue number#05850/05896). The appearance of characteristic cell clusters or colonies in the culture dish was observed daily. Colonies were counted 12-28 days after transduction. iPSCs were harvested, plated onto matrigel-coated plates with mTeSR1 medium, dissociated into small clusters with 1 U/ml dispase (STEMCELL Technologies, Inc.) and subcultured every 4-6 days at a rate of 1:4.

*Differentiation of iPSCs into hepatocytes and experimental grouping.*Hepatic differentiation was performed by using previously described protocols with modififications 17. DMEM/F12 medium and Matrigel were successively added into a 50-ml tube, then the mixture was evenly added into each well of six-well plates. The plates were placed at 4˚C for 1 h, and the medium containing Matrigel was discarded. The iPSCs derived from urinary derived epithelial cells in mTeSR1 medium containing 4 µM rho associated coiled-coil containing protein kinase 1 inhibitor Y27632 were placed in a six-well culture plate at a density of 1.4x10^5^ cells/cm^3^.

On day 2, mTESR1 medium was replaced with definitive endoderm (DE) medium, which was replaced daily for 3 days. DE medium was as follows: RPMI-1640 (gibco; Thermo Fisher Scientific, Inc. catalogue number#11875-093) + B27 Supplement + 100 U/ml penicillin/streptomycin (Thermo Fisher Scientific) + 100 ng/ml activin A (Novus Biologicals) + 3 μM CHIR99021 (Sigma-Aldrich; Merck KGaA).

On day 5, DE medium was replaced with hepatic endoderm (HE) medium, which was changed daily for 5 days. HE medium was as follows: RPMI-1640 + B27 Supplement + 100 U/ml penicillin/streptomycin + 5 ng/ml basic fibroblast growth factor Novus Biologicals) + 20 ng/ml bone morphogenetic protein 4 (Novus Biologicals) + 0.5% DMSO (Sigma-Aldrich; Merck KGaA).

On day 10, HE medium was replaced with immature hepatocyte (IMH) culture medium, which was changed daily for 5 days. IMH culture medium was as follows: RPMI-1640 + B27 Supplement + 100 U/ml penicillin/streptomycin + 20 ng/ml hepatocyte growth factor (HGF; Novus Biologicals) + 0.5% DMSO.

On day 15, IMH medium was replaced with mature hepatocyte (MH) culture medium, which was changed daily for 10-12 days. MH culture medium was as follows: SingleQuots (Lonza Group, Ltd.) + hepatocyte basal medium (Lonza Group, Ltd. catalogue number#CC-3197) + 20 ng/ml HGF + 20 ng/ml Oncostatin M (Novus Biologicals) + 100 nM dexamethasone + 0.5% DMSO. At the end of this stage, mature hepatocytes were obtained.

According to whether Cx32 expression was regulated, upregulated (VK2), downregulated (2-APB) and control groups were established. On the 10th day of hepatic differentiation from iPSCs, the upregulated group was treated with 60 µM VK2 (Sigma-Aldrich; Merck KGaA) to increase Cx32 expression, while the downregulated group was treated with 60 µM 2-APB (Sigma-Aldrich; Merck KGaA) to inhibit Cx32 expression. The control group was not treated with the regulatory factor. Picture of schematic timeline for hepatocyte differentiation of human iPS cells.

*Identification of iPSCs and their directionally differentiated hepatocytes by immunofluorescence staining.* The iPSCs and their directionally differentiated hepatocytes were digested with 2 ml preheated 0.25% trypsin for 2-3 min. The reaction was then terminated and the cells were harvested by centrifugation at 200 x g for 5 min in room temperature. Cells were suspended in an appropriate amount of culture medium and plated into a 12-well plate with a cell slide. Cell growth on the cell slide was observed under a microscope. The cell slides were taken out and put in a 35-mm or 60-mm cell culture dish, fixed with 4% formaldehyde for 10 min, and then washed three times with PBS. Afterwards, cells were incubated with the primary monoclonal anti-mouse antibodies (Invitrogen; Thermo Fisher Scientific, Inc. 1:500 dilution): Cx32 catalogue number#35-8900, Nanog catalogue number#53-5751-80, OCT3/4 catalogue number#MA1-104, hepatocyte nuclear factor 4α(HNF-4α) catalogue number#MA1-199, ALB catalogue number#MA1-19174, cytokeratin 18(CK18) catalogue number#MA1-06326, AFP catalogue number#MA5-14666 that were diluted in 50 mM Tris-HCl, pH 7.4, 1.5% sodium chloride and 0.3% Triton X-100 (TBSTr) supplemented with normal donkey serum(Novus Biologicals) overnight at 4˚C, and incubated with the corresponding Alexa Fluor 488 (green)-conjugated or Alexa Fluor 594 (red)-conjugated secondary donkey anti-mouse antibodies (Invitrogen; Thermo Fisher Scientific, Inc. 1:1000 dilution, catalogue number#A-21202 and A-21203 respectively) for 1 h at room temperature (25˚C). The cells were washed four times with TBSTr and stained with DAPI (Thermo Fisher Scientific, Inc.). Fluorescent signals were examined using an Olympus Fluoview IX70 confocal microscope (Olympus Corporation) and the images were obtained using Olympus Fluoview 2.1 software.

*Reverse transcription-quantitative PCR (RT-qPCR).* Total RNA was extracted from the hepatocytes of different treatment groups according to the instructions of the TrizolTM reagent (Invitrogen; Thermo Fisher Scientific, Inc.), and the RNA concentration was measured using a Nanodrop instrument (Thermo Fisher Scientific, Inc.). Reverse transcription was performed using a SuperScript™ III FirstStrand Synthesis System Kit (Invitrogen; Thermo Fisher Scientific, Inc.). The RT²SYBR Green Fluor qPCR MasterMix Kit (Invitrogen; Thermo Fisher Scientific, Inc.) was used for RT-qPCR. The PCR reaction conditions were as follows: Initial denaturing at 94˚C for 10 min; 40 cycles of denaturation at 94˚C for 15 sec, annealing at 58˚C for 40 sec and extension at 72˚C for 40 sec; plate reading at 81˚C for 1 sec; and annealing curve from 60˚C to 95˚C, every 0.5˚C for 1 sec, and 72˚C for 2 min. GAPDH was selected as a housekeeping gene. The sequences of the primers are shown in Table 1. The single-band PCR product was validated by electrophoresis on a 2% agarose gel stained with ethidium bromide.

*Western blotting.* The hepatocytes in different treatment groups were lysed in immunoprecipitation lysis buffer (20 mM Tris-HCl, pH 8.0, 140 mM sodium chloride, 1% Triton X-100, 10% glycerin, 1 mM EGTA, 1.5 mM magnesium chloride, 1 mM dithiotreitol, 1 mM phenylmethyl sulfonyl fluoride, and 5 μg/ml leptidin, pepsinstatin A and aprotinin), followed by centrifugation at 20,000 x g for 5 min at room temperature to obtain supernatants. The concentration of total protein in supernatants was determined using a Nanodrop instrument (Thermo Fisher Scientific, Inc.). The protein extracts (10 μg per lane) were isolated by SDS-PAGE using a 10 or 12.5% gel and then transferred to a PVDF membrane in a standard Tris-Glycine transfer buffer (pH 8.3). The membrane was blocked at room temperature with 10 mM Tris-HCl, pH 8.0, 150 mM NaCl and 0.2% Tween 20 (TBSTw) containing 5% skimmed milk for 2 h, and incubated with primary antibodies (Invitrogen; Thermo Fisher Scientific, Inc. 1:1000 dilution): Cx32 catalogue number#35-8900, Nanog catalogue number#53-5751-80, OCT3/4 catalogue number#MA1-104, hepatocyte nuclear factor 4α(HNF-4α) catalogue number#MA1-199, ALB catalogue number#MA1-19174, cytokeratin 18(CK18) catalogue number#MA1-06326, AFP catalogue number#MA5-14666 overnight at 4˚C.The next day, the membrane was washed with PBSTw for 40 min and then incubated with the secondary antibody (Invitrogen; Thermo Fisher Scientific, Inc. 1:3,000-1:5,000 dilution, catalogue number#SA1-100) at room temperature. Protein bands were analyzed using a gel electrophoresis apparatus and visualized using enhanced chemical luminescence in the chemiluminescence imaging system.

*Statistical analysis.* Data are presented as the mean and standard deviation. Statistical analysis was performed using GraphPad Prism 7 software (GraphPad Software, Inc.). The difference between three groups was compared using one-way ANOVA analysis, and post hoc test was Tukey test. P<0.05 was considered to indicate a statistically significant difference. For all statistics, data from at least three independent repeated experiments were used.

Three biological replicates were performed for these experiments.

## Results

**Figure 1:**
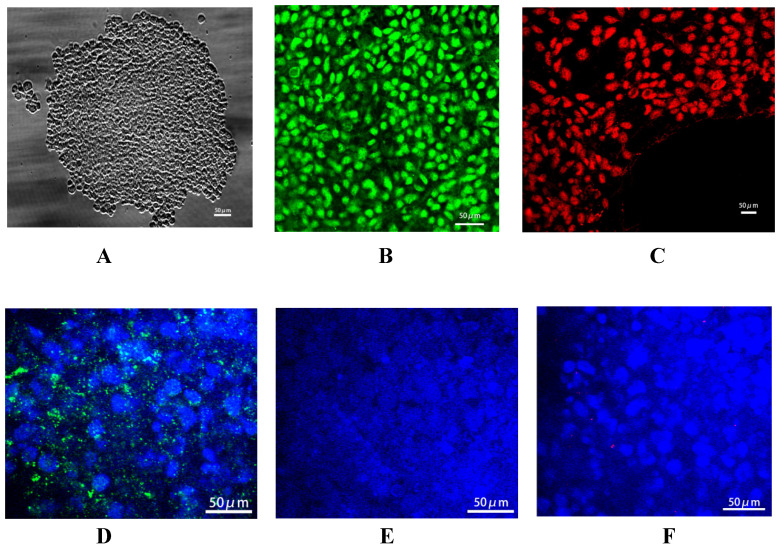
Identification of urine-derived induced pluripotent stem cells (iPSCs). A: Small cell clusters formed by cell colonies. Confocal microscopy showed that iPSCs generated by renal tubular epithelial cells had the morphology of stem cells. B: Nanog expression (green). C: OCT-3 expression (red). D: Cx43 expression (green) in the iPSCs, and DAPI-stained nucleus (blue). E: No AFP expression (red) in the iPSCs, and DAPI-stained nucleus (blue). F: No Cx32 expression (green) in the iPSCs, and DAPI-stained nucleus (blue). Magnification ×400.

*Identification of urine-derived iPSCs.* The reprogramming plasmid mixture was transduced into the urinary derived epithelial cells. After culture, the formation of cell colonies was observed, and these were dissociated into small clusters by dispase (Fig. 1A). Immunofluorescence staining demonstrated the positive expression of specific markers of stem cells, including Nanog (green fluorescence) and OCT-3 (red fluorescence) (Fig. 1B and C), In addition, it showed that these urine derived iPSCs can be used to generate embryoid bodies (EBs) compromising the three germ layers (Suppl Fig. 1). Above all indicated that iPSCs were successfully generated. In addition, connexin 43 (Cx43) is the specific connexin of stem cells, and the expression patterns of connexins in embryonic liver undergo lineage stage-dependent changes during the hepatic differentiation and maturation process. There is a switch from Cx43 to Cx32 expression during the differentiation of hepatic progenitor cells into hepatocytes. In the present study, Cx43 (Fig. 1D) expression was detected after transduction of reprogramming plasmid mixture, while the expression of hepatic specific markers, including Cx32 (Fig. 1E) and AFP (Fig. 1F), was not observed, confirming the generation of iPSCs.

**Figure 2:**
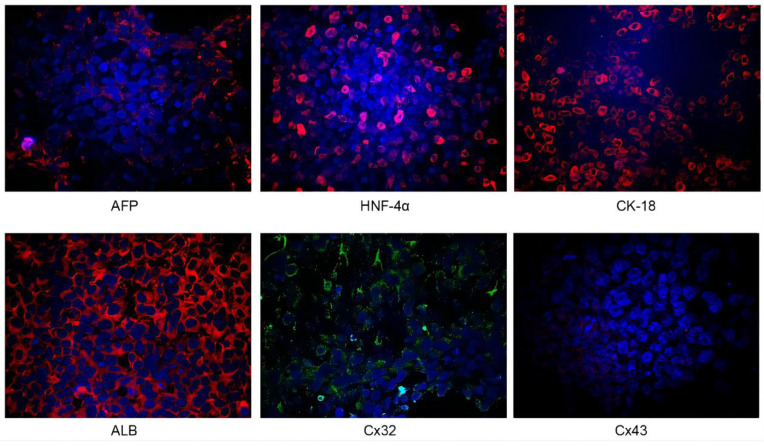
Identification of iPSC-derived hepatocytes. After completion of cell differentiation, the specific markers of hepatocytes, including CK-18, HNF-4α, ALB and AFP were expressed. Meanwhile, the expression of Cx32 but not Cx43 was detected. Magnification ×40.

**Figure 3:**
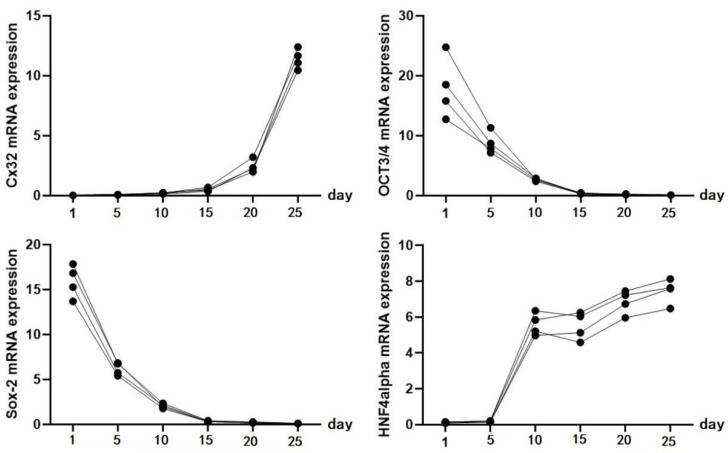
The dynamic mRNA changes of *GJB1* (*CX32*), *POU5F1* (*OCT3/4*), *SOX2,* and *HNF4A* expression was detected during the differentiation from iPSCs to hepatocytes.

*Identification of iPSC-derived hepatocytes.* After the completion of cell differentiation, the hepatic specific markers, including CK18, HNF-4α, ALB and AFP, were positively expressed (Fig. 2). Additionally, the expression of Cx32 but not Cx43 was detected (Fig. 2). Furthermore, the dynamic mRNA changes *GJB1* (*CX32*), *POU5F1* (*OCT3/4*), *SOX2* and *HNF4A* were detected during the differentiation from iPSCs to hepatocytes. The results demonstrated that the mRNA levels of *POU5F1* (*OCT3/4*) and *SOX2* were gradually decreased and those of *GJB1* (*CX32*) and *HNF4A* were gradually increased with the extension of differentiation time (Fig. 3). These data suggested successful differentiation from iPSCs to hepatocytes.

**Figure 4:**
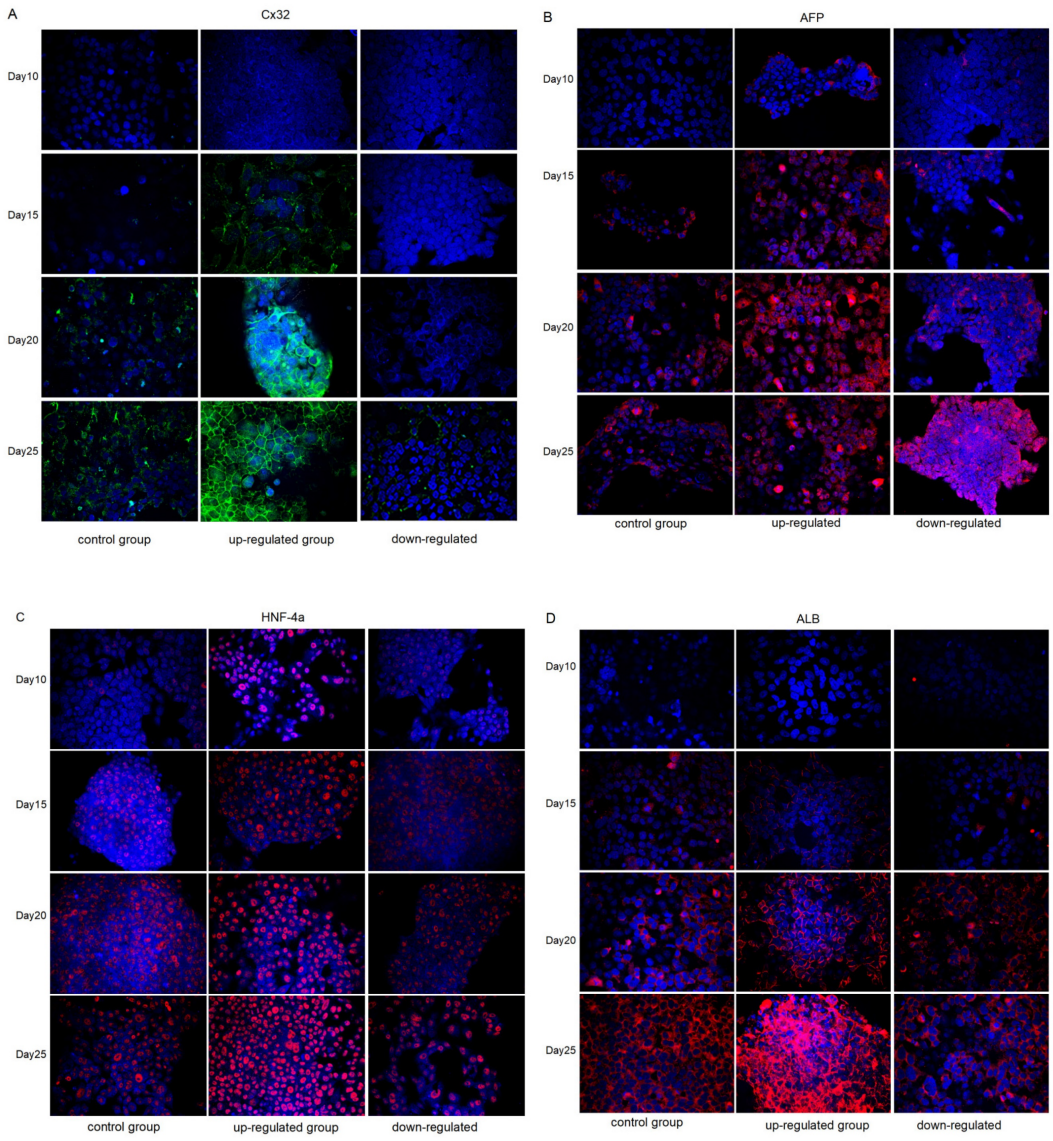
Immunofluorescence analysis of the expression changes of Cx32, AFP, HNF-4α, and ALB in control groups, VK2 treatment group (Cx32 up-regulated) and 2-APB treatment group (Cx32 down-regulated) during the differentiation from iPSCs to hepatocytes. A: Cx32 expression (green). B: AFP expression (red). C: HNF-4α expression (red). D: ALB expression (red). Magnification ×400.

Immunofluorescence analysis of the expression changes of Cx32, AFP, HNF-4α and ALB in different groups during the differentiation from iPSCs to hepatocytes. In the control group, marked Cx32 expression was observed until the 20th day of differentiation. Cx32 was markedly expressed on the 15th day of differentiation in the upregulated group, while Cx32 was still slightly expressed on the 25th day of differentiation in the downregulated group. These data indicated that, compared with that in the control group, Cx32 expression appeared earlier and markedly increased in the upregulated group, while the opposite effect was observed in the downregulated group (Fig. 4A).

The expression of the hepatic specific markers, including AFP (Fig. 4B), HNF-4α (Fig. 4C) and ALB (Fig. 4D), was further analyzed during the differentiation from iPSCs to hepatocytes. Compared with that in the control group, AFP expression appeared earlier and markedly increased in the upregulated group; however, at the later stage of differentiation, AFP expression showed a weakening trend unlike Cx32, suggesting that hepatocytes matured earlier in the upregulated group than in the control group. In the downregulated group, AFP expression appeared later and was markedly increased in the later stage of differentiation, suggesting that the hepatocyte maturation was delayed in the downregulated group. In addition, compared with that in the control group, the expression of HNF-4α and ALB appeared earlier and was markedly increased in the upregulated group, and the opposite effects were observed in the downregulated group.

**Figure 5:**
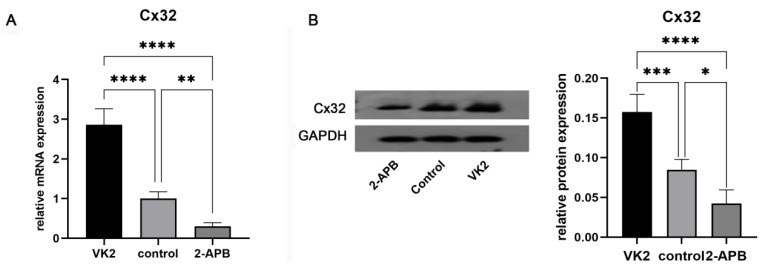
Analysis of Cx32 expression in different groups after the differentiation was completed. A: qRT-PCR showed the *GJB1 RNA* (*Cx32*) level in different groups. B: Western blot showed the Cx32 protein expression in different groups. The error bars in Figure 6B represent standard deviation. GAPDH was used as an internal control. * p < 0.05 compared to the control group.

*Analysis of Cx32 expression in different groups.* After the differentiation was completed, *GJB1 RNA* (*Cx32*) and Cx32 protein expression in different groups was determined by RT-qPCR and western blotting, respectively. As shown in Fig. 5, both the mRNA and protein expression in the upregulated group was markedly higher than that in the control group, while these in the downregulated group was markedly lower.

**Figure 6:**
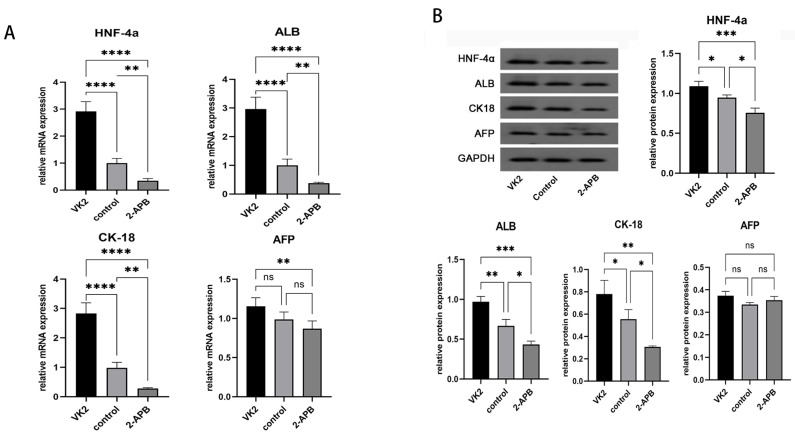
Analysis of the expression of the specific markers of hepatocytes in different groups after the differentiation was completed. A: qRT-PCR showed the mRNA expression of *HNF4A, ALB, KRT18* (*CK18*), and* AFP* in different groups. B: Western blot showed the protein expression of HNF-4α, ALB, CK18, and AFP in different groups. The error bars represent standard deviation. GAPDH was used as an internal control. * p < 0.05 compared to the control group.

*Analysis of the expression of the specific markers of hepatocytes in different groups.* After the differentiation was completed, the mRNA and protein expression levels of hepatic specific markers, including HNF-4α, ALB, CK18 and AFP, were also detected. The results demonstrated that, compared with the control group, both the mRNA and protein expression levels of specific markers of hepatocytes, including HNF-4α, ALB, CK18 and AFP, exhibited marked increases in the upregulated group, while these were markedly decreased in the downregulated group (Fig. 6).

## Discussion

Hepatocyte transplantation is seriously hindered by the insufficient resource of viable donor hepatocytes, difficulties in cryopreservation, inability of proliferation and de-differentiation *in vitro.* Stem cell-based approaches, especially iPSCs, could help solve the organ shortage in transplantation, offering a novel approach for cell transplantation.

In the present study, iPSCs were successfully generated from human urine-derived renal tubular epithelial cells by reprogramming. The iPSCs were directionally differentiated into mature hepatocytes. Furthermore, Cx32 expression was not observed in iPSCs but gradually increased during the process of hepatic differentiation from iPSCs. Additionally, upregulation of Cx32 promoted hepatocyte maturation and enhanced the expression of hepatic specific markers, whereas downregulation of Cx32 had the opposite effects. Taken together, it could be hypothesized that Cx32 was a key regulatory factor during the process of hepatic differentiation from iPSCs.

Several types of stem cells, including ESCs, mesenchymal stem cells (MSCs) and iPSCs, have the capacity of self-renewal and are able to be differentiated into hepatocyte-like cells, which have aroused much attention as alternative hepatic cell sources to study liver diseases. However, ethical issues, immune rejection in hESCs^18^, and variable proliferation rate, differentiation potential and immune regulation in MSCs^19^,^20^ have hindered their application. In 2006, iPSCs were successfully generated, exhibiting the characteristics of ESCs 21, and have become a major breakthrough in the field of cell and tissue engineering transplantation. Multiple types of somatic cells can be reprogrammed into iPSCs via nuclear transfer and transcription factor-based reprogramming, and urinary derived epithelial cells have been considered as desirable cell sources because they are conveniently, non-invasively and cost-effectively obtained from urine 22,23. The iPSCs generated from human urine can be differentiated into hepatocytes for transplantation, which can solve the problem of insufficient cell sources.

The directional differentiation of iPSCs into hepatocytes is mainly based on the differentiation strategy of hESCs 24. To generate hepatocyte-like cells, the present study adopted the directional differentiation protocol published by Cowan's group 25,26. Since human-derived iPSCs were first efficiently induced to differentiate into hepatocyte-like cells *in vitro* by Song *et al.* 27, more laboratories have proposed a variety of optimization schemes, such as using hypoxia in the middle stage of differentiation to improve differentiation efficiency 7, improving the culture substrates 28, replacing cytokines with small molecular compounds 29 and applying tissue engineering technology to form three-dimensional organoids 30. These studies have improved the differentiation efficiency and functional maturity of iPSC-derived hepatocytes to a certain extent, but iPSC-derived hepatocytes usually carry fetal hepatocyte-like phenotype and functions 31 and exhibit certain differences compared with primary cultured mature hepatocytes. To improve the directional differentiation efficiency and functional maturity of iPSC-derived hepatocytes, it is still necessary to explore the regulatory factors during the process of hepatic differentiation from iPSCs.

Connexins are a family of transmembrane proteins that form gap junctions to mediate cell-cell communication and serve a regulatory role in maintaining the homeostasis, function and metabolism of the intracellular environment 32,33. The expression of the connexin family is tissue-specific, and Cx32 is a major gap junction protein in the liver and brain 34. Cx32 expression is associated with most liver-specific functions such as ammonia detoxification, ALB secretion, glycogenolysis and bile secretion 35,36. Cx32 accounts for ~90% of connexins on hepatic parenchymal cells 37, which is crucial for maintaining the differentiation, maturation and specific phenotypes of hepatocytes 38,39. Accumulating evidence has revealed that Cx32 has a protective effect on hepatic homeostasis. Cx32 knockout mice are more susceptible to acetaminophen-induced hepatotoxicity compared with wild-type mice 40, there is more pronounced liver damage, inflammation and oxidative stress in mice with Cx32 deficiency 41, and transgenic rats with a dominant-negative mutant of Cx32 dominant-negative have been found to be resistant to hepatic damage by chemicals, such as D-galactosamine and carbon tetrachloride 42. The amounts of Cx32 protein are markedly decreased in acute drug-induced hepatotoxicity, fibrosis, hepatitis, cirrhosis and hepatocellular carcinoma 43. Most importantly, the expression patterns of connexins undergo lineage stage-dependent transformation in the embryonic liver. Cx32 was not expressed in the early undifferentiated stage of iPSCs, but its expression was increased gradually with the differentiation into hepatocytes, suggesting that this protein may exert an active regulatory function during the differentiation process. Avior *et al.* 44 demonstrated that VK2 promoted the maturation of hepatocytes via increasing Cx32 expression. Qin *et al.* 45 also revealed that VK2 upregulated Cx32 expression to promote hepatic differentiation and maturation from hESCs 16, while the negative Cx32 regulator 2-APB had the opposite effect. These findings all suggest the positive role of Cx32 in the differentiation of ESCs into hepatocytes. In addition, current data have indicated that there is no significant difference in the hepatic differentiation abilities of hESCs and iPSCs 7, and iPSCs as a cell source for hepatic differentiation may be more valuable than hESCs 37.

The present study preliminarily explored the role of Cx32 in the differentiation and maturation of hepatocytes from iPSCs; however, the possible regulatory mechanism of Cx32 remained unclear. Some studies have revealed that the p38 MAPK signaling pathway, which is associated with Cxs, may be involved in the regeneration of rat hepatocytes 46. The bioinformatics data suggested that both SB (p38 MAPK inhibitor) and VK2 might affect the hepatic differentiation process in similar ways through negative regulation of the p38 signaling MAPK pathway 16. As for 2-APB, it suggested that 2-APB inhibit connexin channels by acting at the intracellular side of the connexin, the potential sites is segment of the C-terminal domain closest to the membrane that is involved in 2-APB inhibition 47. Thus, more studies are required to elucidate the molecular and pathway mechanisms of Cx32 in hepatic differentiation. There are some limitations of the present study. For example, VK2 and 2-APB may have a potential non-specific effect when regulating Cx32 expression, thus targeting the Cx32 gene via genetic methods could produce more convincing evidence. Additionally, in the experiment of validating hepatic function, although hepatocyte-specific markers, including ALB, AFP, HNF-4a and Cx32, can roughly reflect hepatic function, more markers, such as thrombinogen, apolipoprotein, cytochrome P450, glycogen, urea and others, should be tested to provide stronger verification. However, due to the tight budget, we only selected the most typical markers (HNF-4a, CK18, ALB, and AFP) and verified their production during the differentiation process. We believe they could roughly reflect the functionalities of differentiated hepatocytes. If possible, we would make further research in this field.

In conclusion, the present research conducted the whole process from urine-derived cells to iPSC, further to hepatocyte-like cells, that no other research has done such complete and prolonged process. Thus, it revealed that urine-derived iPSCs could be directionally differentiated into mature hepatocytes. Upregulation of Cx32 improved the of differentiation of iPSC into hepatocytes. Cx32 may be a promoting factor during the directional differentiation of iPSCs into mature hepatocytes. The findings of the present study will provide an experimental basis for hepatocyte transplantation. More importantly, the iPSCs could be generated from the renal tubular epithelial cells in the patient's own urine, which will avoid immune rejection caused by genetic differences to a certain extent. Whereas limitation still exist, that is it aims to conclude that the role of Cx32 is to promote differentiation and maturation of hepatocytes from iPSCs, rather than its mechanism. Although it was widely acknowledged that specific connexin form channels to permit rapid intercellular exchange of small signal molecules, thus resulting cellular activities, including the regulation of growth, differentiation, and developmental signaling, but the concrete functional molecules and signal pathways have not been elucidated. Therefore, further clinical studies are required to confirm the present findings.

## Author contributions

Study design: XL, BS; Data collection: RQ, KL; Analysis and interpretation: YX, XW; Statistical analysis: YW; Drafting manuscript: YX, YW; Revision manuscript: XL, YX. All authors contributed to manuscript revision, read, and approved the submitted version.

## Ethics statement human experimentation

The studies involving human participants were reviewed and approved by the Ethics Committee of Tongji Hospital Affiliated to Tongji University, approval number: K-2022-029.

## Informed consent statement

The participants provided their written informed consent to participate in this study and publish this paper.

## Data availability statements

The original contributions presented in the study are included in the article material, further inquiries can be directed to the corresponding authors.

## Figures and Tables

**Table 1:** The primer sequences used in this study

Gene	Primer sequences	
	Forward (5'-3')	Reverse (5'-3')
*Cx32*	TCCCTGCAGCTCATCCTAGT	TGAGATGTGGACCTTGTGCC
*ALB*	CCTTTGGCACAATGAAGTGGGTAACC	CAGCAGTCAGCCATTTCACCATAGG
*AFP*	AGAACCTGTCACAAGCTGTG	GACAGCAAGCTGAGGATGTC
*CK18*	AAATCCGGGAGCACTTGGAG	CAATCTGCAGAACGATGCGG
*HNF-4α*	CTGCTCGGAGCCACCAAGAGATCCATG	ATCATCTGCCAGGTGATGCTCTGCA
*GAPDH*	GCCAAGGTCATCCATGACAAC	GTCCACCACCCTGTTGCTGTA
OCT3	CTCGAGAAGGATGTGGTCCG	AGCCTGGGGTACCAAAATGG
